# ComPlEx: conservation and divergence of co-expression networks in *A. thaliana*, *Populus* and *O. sativa*

**DOI:** 10.1186/1471-2164-15-106

**Published:** 2014-02-06

**Authors:** Sergiu Netotea, David Sundell, Nathaniel R Street, Torgeir R Hvidsten

**Affiliations:** 1 Umeå Plant Science Center, Department of Plant Physiology, Umeå University, Umeå, Sweden; 2 Computational Life Science Cluster (CLiC), Umeå University, Umeå, Sweden; 3 Department of Chemistry, Biotechnology and Food Sciences, Norwegian University of Life Sciences, P.O. Box 5003, Ås NO-1432, Norway

## Abstract

**Background:**

Divergence in gene regulation has emerged as a key mechanism underlying species differentiation. Comparative analysis of co-expression networks across species can reveal conservation and divergence in the regulation of genes.

**Results:**

We inferred co-expression networks of *A. thaliana*, *Populus spp.* and *O. sativa* using state-of-the-art methods based on mutual information and context likelihood of relatedness, and conducted a comprehensive comparison of these networks across a range of co-expression thresholds. In addition to quantifying gene-gene link and network neighbourhood conservation, we also applied recent advancements in network analysis to do cross-species comparisons of network properties such as scale free characteristics and gene centrality as well as network motifs. We found that in all species the networks emerged as scale free only above a certain co-expression threshold, and that the high-centrality genes upholding this organization tended to be conserved. Network motifs, in particular the feed-forward loop, were found to be significantly enriched in specific functional subnetworks but where much less conserved across species than gene centrality. Although individual gene-gene co-expression had massively diverged, up to ~80% of the genes still had a significantly conserved network neighbourhood. For genes with multiple predicted orthologs, about half had one ortholog with conserved regulation and another ortholog with diverged or non-conserved regulation. Furthermore, the most sequence similar ortholog was not the one with the most conserved gene regulation in over half of the cases.

**Conclusions:**

We have provided a comprehensive analysis of gene regulation evolution in plants and built a web tool for *Com*parative analysis of *Pl*ant co-*Ex*pression networks (ComPlEx, http://complex.plantgenie.org/). The tool can be particularly useful for identifying the ortholog with the most conserved regulation among several sequence-similar alternatives and can thus be of practical importance in e.g. finding candidate genes for perturbation experiments.

## Background

A functional role has been ascribed to only about half of all plant protein coding genes to date. For a given species the majority of functional information is typically transferred from *Arabidopsis thaliana* orthologs identified by sequence similarity searches. However, due to the existence of large gene families in plants, these searches frequently identify several alternative orthologs for each gene. Adding to this complexity, gene function can often only be understood in the context of other genes (as emergent properties) [1,2], and accumulating evidence suggests that divergence in gene regulation rather than the protein coding sequence is the main driving force behind species differentiation [1,3,4]. Therefore, a powerful and appealing approach to studying gene function across species is to combine traditional methods based on individual genes and static sequence information (comparative genomics) with network-based methods that incorporate dynamic omics data (comparative regulomics).

Gene expression databases have been expanding rapidly since the first high-throughput microarray studies were published in the 1990s [5,6]. Resources such as the Gene Expression Omnibus [7] now enable us to compile datasets from several species that extensively profile gene expression dynamics across large panels of stress conditions, developmental gradients, tissues and genotypes. Hence, we are now, for the first time, able to extensively compare gene regulation across multiple species. Cross-species analysis of gene regulation can be achieved by directly comparing expression profiles or by indirectly comparing co-expression clusters or networks [8]. Direct comparison of profiles requires gene expression to be quantified in comparable samples or tissues in two or more species (e.g. [9-11]). For example, Patel *et al.*[11] identified orthologs with the most correlated expression profiles across equivalent tissues (“expressologs”) in seven plant species. Comparison of co-expression across species, on the other hand, examines to what degree co-expressed genes in one species are also co-expressed in another species, and thus does not depend on comparable samples (e.g. [12,13]). For example, Yim *et al.*[13] computed gene function enrichment in lists of co-expressed genes and compared the results across eight plant species.

A general approach to cross species comparison of gene regulation is that of network alignment, that is, to compare co-expression networks by constructing a map connecting nodes (i.e. genes) across the networks (i.e. species). As is the case for sequence alignment, network alignment methods can produce both local and global alignments between two or more species. Furthermore, network alignments can map either individual genes or entire modules of highly connected genes [14], and the map can be purely ortholog-based (i.e. based on sequence similarity) [14,15], purely topology-based (i.e. based on network similarity) [16] or a combination of the two [17-21]. Network alignment methods were initially developed for protein interaction networks [22]. Some of these methods incorporated models of protein network evolution [23], while such models have only recently been proposed for transcriptional networks [4].

In plants, three particularly interesting network-based studies of co-expression conservation have been published (for a review see Movahedi *et al*. [24]). Mutwil *et al.*[25] computed the similarity of co-expression network vicinities based on Pfam [26] across seven plant species. The method has also been used to construct a consensus co-expression network for cellulose synthase (CESA) genes involved in secondary cell wall formation [27]. Mohavedi *et al*. [28] computed the similarity between co-expression network neighbourhoods, based on the expression context conservation (ECC) score, in *A. thaliana* and *Oryza sativa*. Both approaches were purely ortholog-based. Ficklin *et al.*[29], however, used a network alignment method called IsoRankN [21], which combines both ortholog and topology information, to align co-expression networks of *O. sativa* and *Zea mays*.

Beyond comparing gene-gene links and network neighbourhoods directly, methods have recently been developed to examine global and local properties of networks to gain insight into their evolution. One global property of biological networks is that they tend to be scale-free, that is, the distribution of the number of links (i.e. neighbours) per node follows a power law [30-32]. A consequence of this is that most nodes have few neighbours while a few nodes (called hubs) have many neighbours. Networks can also be used to identify central genes. Genes with many neighbours have high *degree centrality* and genes with neighbours that have many neighbours have high *average nearest neighbour centrality*. These are both examples of genes with high local centrality, while genes that often are part of the shortest route between two arbitrary genes in the network are examples of genes with high global centrality (*betweenness centrality*). Another property of biological networks is that they are modular and consists of sparsely connected network motifs [33,34]. Network motifs are recurring patterns of links between a small number of nodes (e.g. the feed-forward loop), and it has been suggested that significantly recurring motifs are templates used to realize particular functions effectively and that networks partially evolve through reuse of such motifs [35]. Several of these network properties was described in a co-expression network of *A. thaliana*[36].

While previous comparative studies in plants have revealed interesting properties of gene expression conservation, these studies have not utilized recent advancements in network-based analysis. In particular, network properties, such as gene centrality and network motifs, have not been investigated. Here we present a comprehensive study of *A. thaliana*, *Populus spp.* and *O. sativa* co-expression networks that compares network properties and motifs as well as co-expression links and neighbourhoods. Furthermore, previous studies inferred networks using Pearson correlation, a measure that has numerous issues [37], and only compared networks at a fixed co-expression threshold. We conduct our analysis over a range of co-expression thresholds and infer networks using mutual information (MI) and context likelihood of relatedness (CLR), an approach shown to be state of the art in comparative studies [38,39]. As a result, our approach yielded several novel biological observations. For example, we show that scale free characteristics in the three species only emerge above a certain co-expression threshold and that high centrality of genes tends to be conserved. While relatively few gene-gene co-expression links are conserved across the species, network neighbourhoods are largely conserved statistically, especially when comparing networks at low co-expression thresholds where the statistical power is greater. Finally, by integrating ortholog and network topology information, we show that, for genes with more than one predicted ortholog, in over half the cases the most sequence similar ortholog is *not* the one with the most conserved gene regulation. We also present a web tool for *Com*parative analysis of *Pl*ant co-*Ex*pression networks (ComPlEx) that offers flexible analysis of network conservation in plants.

## Results

### Network inference and comparison

We retrieved all available Affymetrix gene expression microarray data for *A. thaliana* (At, 6,665 experiments, 19,115 genes, 1,308 transcription factors), *Populus* (Pt, 462 experiments, 27,793 genes, 1,870 transcription factors) and *O. sativa* (Os, 711 experiments, 15,470 genes, 957 transcription factors) from the Gene Expression Omnibus (GEO) [40]. From the expression data of each species, co-expression networks were inferred by computing mutual information (MI) for each gene pair, applying the context likelihood of relatedness (CLR) algorithm to obtain background-corrected Z-score and finally by applying a threshold to decide whether a gene-pair should be linked or not (henceforth referred to as the CLR threshold). In our analysis, we considered both *gene co-expression networks* containing all undirected links above a certain CLR threshold and *gene regulation networks* containing all directed links above a certain CLR threshold going from a transcription factor (TF) to a gene. In the gene regulation networks the links are interpreted as putative regulation where TFs physically regulate genes through binding to DNA.

**Figure 1:**
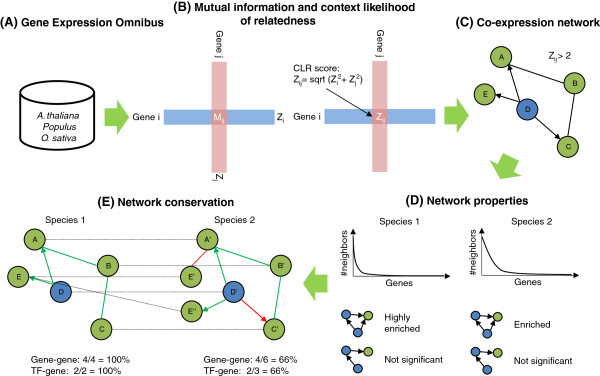
**Method overview. (A)** Gene expression data for the three plants were downloaded and **(B)** used to compute the mutual information and the corresponding CLR score for each gene pair. **(C)** A co-expression network was constructed by connecting all gene pairs with a CLR score above a certain threshold. Links from transcription factors (TFs, blue) to genes (green) were represented as directed links (arrows). Networks from different species were then compared at different CLR thresholds with respect to **(D)** network properties and network motifs) and **(E)** fraction of conserved links or conserved neighbourhoods.

We considered genes in the same OrthoMCL group [41] to be *predicted orthologs* (if from different species) and *predicted paralogs* (if from the same species). The average number of predicted orthologs per gene varied from two to four for different species-pairs. For example, a *Populus* gene had on average 2.8 predicted orthologs in *A. thaliana*, while an *A. thaliana* gene had 3.6 predicted orthologs in *Populus* (Additional file 1: Figure S1). Using predicted orthologs, we compared networks across the three plant species based on network properties (e.g. betweenness centrality), network motifs (e.g. feed-forward loops), conservation of gene-gene co-expression links and conservation of network neighbourhoods. A conceptual outline of the methodology is represented in Figure 1 (see Methods for details).

We also constructed a web portal for *Com*parative analysis of *Pl*ant *Ex*pression networks (ComPlEx: http://complex.plantgenie.org/). The site allows users to explore and compare subnetworks of the three plant species, and provides complete results and associated statistics for all the results presented in this article.

### Network properties

**Figure 2:**
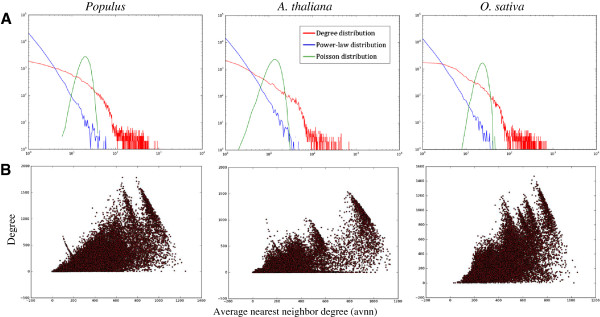
**Network properties. (A)** Comparison of node degree distributions in the gene regulation networks at a CLR threshold of four. The degree distribution is fitted to a power-law distribution and a Poisson distribution with the same statistical parameters. **(B)** Average nearest neighbour degree (X axis) versus degree (Y axis) in the co expression networks at a CLR threshold of four.

We performed an extensive investigation of network properties by comparing degree distributions, gene centralities and subnetwork statistics across species and CLR thresholds (Figure 2).

#### Degree distributions

The degree of a node is the number of links it has to other nodes (number of neighbours). We fitted the degree distributions of our networks to the power-law distribution [30] and the Poisson distribution [42]; the former describes scale-free networks while the latter describes networks where each link occurs independently with an equal probability (commonly referred to as *random networks*). For all species there was a CLR threshold that acted as a state switch; above this threshold the degree distribution followed a power law (i.e. the network was scale-free) while below this threshold we observed an exponential increase in the number of links and network degrees distributions that approached that of random networks (Figure 2A).

#### Gene centralities

Gene centralities indicate the relative importance of genes within a network (see Background). We observed that e.g. degree centrality and average nearest neighbour (avnn) centrality were positively correlated at the global scale (assortative), but tended to be negatively correlated for visually distinct subsets of genes (locally disassortative, Figure 2B); a trend that became more pronounced in the scale free range (i.e. for higher CLR thresholds). In *A. thaliana*, there were two disassortive groups associated with *poly (U) RNA* or *chlorophyll binding* (FDR corrected p-values less than 3E-10 [43]) and *heme binding*/*peroxidase activity* (P < 3E-20), respectively. At the gene level, we found that genes with high local centrality in one species (top 10%) were significantly more likely to also have high centrality in the two other species (Additional file 2). In co-expression networks with a CLR threshold of four, ~29% of genes with high degree centrality in one species, and ~40% of genes with high avnn centrality, also had high centrality in the two other species (P < 1E-4). These genes were enriched for similar biological processes including *photosynthesis* (P = 2.9E-40 for degree centrality and 1.5E-55 for avnn centrality), *generation of precursor metabolites and energy* (P = 5E-20 and 2.5E-24), *translation* (P = 7.9E-5 and 5.9E-5) and *response to abiotic stimulus* (P = 0.02 and 3E-5). High global centrality in terms of betweenness centrality was considerably less conserved across all species (~5%, P = 0.0265) and only enriched for *transporter activity* (P = 0.0092). Unlike in the co-expression networks, degree and avnn centrality displayed markedly different characteristics in the gene regulation networks. High degree centrality was more conserved than high avnn centrality (27% versus 18%, P < 1E-4), and was expectedly associated with regulatory processes (hubs in the regulatory network are typically TFs), but also several developmental processes including *post-embryonic development* (2.0E-8) and the more specific term *flower development* (0.005) as well as *response to endogenous stimulus* (3.6E-13). Genes with high avnn centrality (genes regulated by high degree TFs) were enriched for some of the same processes as in the co-expression network. Unlike high centrality, *low* centrality was usually not significantly conserved in either network types.

#### Subnetwork statistics

We also studied various network statistics within Gene Ontology (GO, [44]) subnetworks, that is, parts of networks containing genes annotated to the same GO category (Additional file 3). We classified a network statistics as conserved in a GO subnetwork if that subnetwork was ranked among the top 10% in all three species. Network density is the ratio between the number of actual links and the number of possible links in a network. In co-expression networks with a CLR threshold of four, high density was strongly conserved across the three species (49%, P < 1E-4) and was expectedly observed in subnetworks with many high degree genes (e.g. *photosynthesis*). Low density was less conserved (21%, P < 1E-4), but included several development-subnetworks (e.g. *embryo development ending in seed dormancy*). The connectivity of a subnetwork is the ratio between the number of links from the subnetwork to the rest of the network and the number of links within the subnetwork [36]. Both high and low connectivity were weakly conserved (6% and 15%, respectively P < 1E-4). Interestingly, subnetworks with conserved low connectivity were all metabolic processes indicating that these constitute separate modules in the networks. In the gene regulation networks we also found that highly regulat*ed* (high ingoing connectivity) and highly regulat*ing* (high outgoing connectivity) subnetworks were conserved (both 16%, P < 1E-4). For example, *protein complex assembly* was highly regulated, while *response to xenobiotic stimulus* and *stem cell/seedling development* were highly regulating, across all species.

### Network motifs

**Figure 3:**
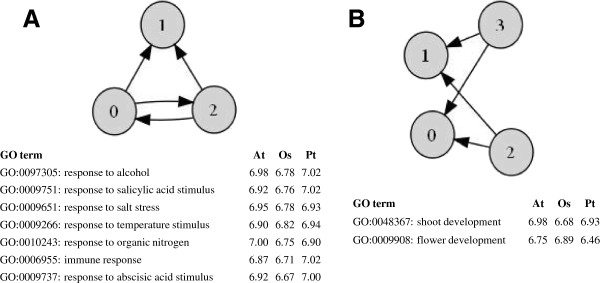
**Network motifs.** The two most prominent motifs found to be significantly conserved across the three species. A selection of Gene Ontology subnetworks were the motif had a top 20% Z-score in all species is listed below each motif (Z-score is given for each species). A an exhaustive list of GOs are given in Additional file 4. **(A)** Two connected transcription factors (TFs) regulating the same gene (feed-forward loop). **(B)** Two unconnected TFs regulating the same two genes (bi-fan motif).

We defined network motifs as directed graphs of three or four genes that were enriched in GO gene regulation subnetworks compared to randomized networks (see Methods). We were specifically interested in motifs that were conserved, that is, highly enriched in the same subnetwork in all three species (top 20%, Additional file 4). At a CLR threshold of four, two out of six possible motifs with three genes, and seven of 22 possible motifs with four genes, were conserved in a significant number of different subnetworks (P < 0.0245). Two particularly interesting motifs were observed: (1) The most conserved motif (41%, P < 1E-4) consisted of two connected TFs regulating the same gene (FFL: feed-forward loop, Figure 3A). This motif also reappeared as part of several highly conserved motifs with four genes. (2) The third most conserved motif (35%, P < 1E-4), and the only motif not containing the FFL pattern among the five most conserved motifs (>20%), consisted of two unconnected TFs both regulating the same two genes (bi-fan motif, Figure 3B). Although these two motifs were also conserved in some common GO subnetworks, their GO profiles were distinctly different. Motif 1 (FFL) was chiefly associated with a number of responses to stimulus while motif 2 was chiefly associated with shoot and flower development (Figure 3).

### Gene-gene co-expression

**Figure 4:**
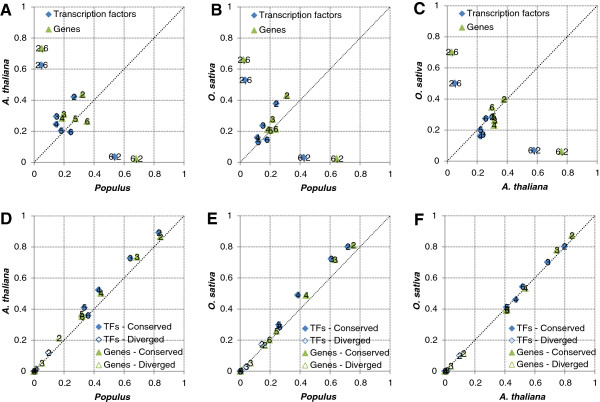
**Network conservation. (A-C)** Gene co-expression (link) conservation and **(D-F)** network neighbourhood conservation. One coordinate (*x*, *y*) corresponds to two fractions of link/neighbourhood conservation (or divergence); *x* is the fraction of conserved links/neighbourhoods when comparing the species on the *x*-axis to the species on the *y*-axis, and *y* is the fraction of conserved links/neighbourhoods when comparing the species on the *y*-axis to the species on the *x*-axis. The numbers in the plot is the CLR-threshold used to construct the compared networks (two CLR thresholds are given when different thresholds were used for the two compared networks). Results are given both for co-expression networks (Genes, green) and gene regulation networks (Transcription factors, blue).

We performed pairwise comparisons of the species where a link between genes A and B in the network of one species was considered conserved if a link existed between any of the orthologs of A and any of the orthologs of B in the network of the other species. Figure 4A-C shows, for CLR thresholds from two to six, the fraction of conserved links in each comparison. The trend for all species was that as the CLR threshold was increased, the fraction of conserved links dropped. However, when we compared networks with a CLR threshold of six against networks with a CLR threshold of two (Figure 4A-C), the fraction of conserved links was dramatically higher than when comparing networks with the same threshold. For all species and thresholds, the conservation observed in co-expression networks was higher than that observed in randomized networks. In fact, at a CLR threshold of two, ~30-40% of the links were conserved even though the networks only contained around 10% of all possible links.

### Network neighbourhoods

We next performed pairwise comparisons of the species where the regulation of a gene was considered conserved if its network neighbourhood (i.e. all genes with a link to it) had a statistically significant overlap with the neighbourhood of one of its orthologs in the other species (see Methods). Figure 4D-F shows, for CLR thresholds from two to six, the fraction of genes with conserved network neighbourhoods in each comparison. Clearly, network neighbourhoods were vastly more conserved than gene-gene links. Even more clearly than for link conservation, neighbourhood conservation was higher for networks with many links; around 80% for a CLR threshold of two and dropping as the CLR threshold increased. Comparing network neighbourhoods across species also allowed us to identify network neighbourhood divergence (i.e. statistically significant underrepresentation of nodes in common between two neighbourhoods). Divergence was primarily observed in large networks (CLR thresholds of two and three) where ~10-20% of the neighbourhoods were significantly diverged (Figure 4D-F).

#### A core of conserved genes

**Figure 5:**
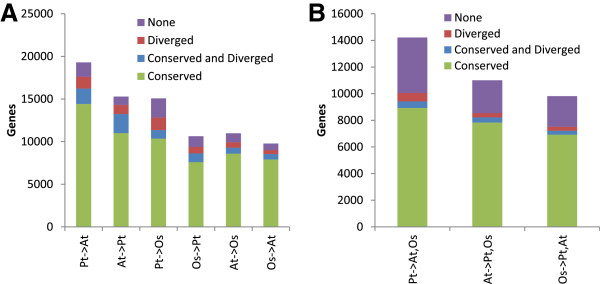
**Number of conserved, diverged and conserved-and-diverged genes in network comparisons. (A)** Pairwise comparisons of network neighbourhoods at a CLR threshold of two. In each comparison X ─ > Y, the genes in species X is divided into genes with at least one conserved ortholog-neighbourhood in species Y (Conserved), genes with at least one conserved and at least one diverged neighbourhood in Y (Conserved and Diverged), genes with at least one diverged neighbourhood in Y (Diverged) and genes with no significant neighbourhoods in Y (None). These are the number of genes behind the fractions plotted in Figure 4D-F (at a CLR threshold of two). **(B)** Corresponding to **(A)** but now each comparison X ─ > Y, Z requires that a gene is e.g. conserved if at least one ortholog-neighbourhood is conserved in both species Y and Z.

While Figure 4D-F shows the *fraction* of genes with conserved and diverged network neighbourhoods for different CLR thresholds, Figure 5A shows the corresponding *number* of conserved/diverged genes for a fixed threshold of two. Since genes had multiple predicted orthologs, we observed a number of genes where at least one of the orthologs had a conserved neighbourhood while at least one of the other orthologs had a diverged neighbourhood. This overlap between conservation and divergence caused the fractions of conserved and diverged genes to sum to more than one in the *Populus*-*A. thaliana* comparisons in Figure 4D. We also calculated how many genes in a given species were conserved/diverged in both the two other species (Figure 5B). This showed that there exists a core of about 8,000 genes (8,223 *A. thaliana* genes) with conserved gene regulation across all three plant species. This conserved core was most enriched for the GO biological process categories *protein modification process* (p = 2.8e-19) and *photosynthesis* (p = 5.4E-10) (Additional file 5: Figure S2). Conversely, there were 704 *A. thaliana* genes with diverged gene regulation in both *Populus* and *O. sativa* and 389 genes with at least one ortholog with conserved regulation and at least one other ortholog with diverged regulation in the two other plants. These were both most enriched for *protein modification process*, *carbohydrate metabolic process* and *pollen-pistil interaction*, but there were also differences such as for *anatomical structure morphogenesis* that was significant (p = 5.1E-04) only for the genes with both conserved and diverged neighbourhoods (Additional file 5: Figure S2).

#### Multiple predicted orthologs

**Figure 6:**
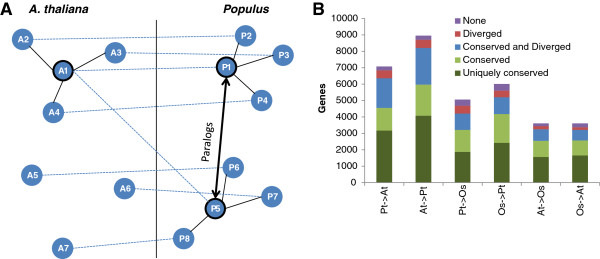
**Genes with multiple predicted orthologs. (A)** Conceptual drawing showing how a gene A1 in *A. thaliana* can have a neighbourhood that is both conserved when compared to one ortholog P1 in *Populus* and diverged if compared to another ortholog P5. Lines between genes in different species indicate orthologs. **(B)** Pairwise comparisons of network neighbourhoods of genes with more than one predicted ortholog at a CLR threshold of two. In each comparison X ─ > Y, the genes in species X is divided into genes where all ortholog-neighbourhoods are conserved in species Y (Uniquely conserved), genes with at least one conserved neighbourhood in species Y (Conserved), genes with at least one conserved and at least one diverged neighbourhood in Y (Conserved and Diverged), genes with at least one diverged neighbourhood in Y (Diverged) and genes with no significant neighbourhoods in Y (None).

We investigated genes with more than one predicted ortholog more thoroughly (Figure 6). About 45% of the genes only had orthologs with conserved regulation (i.e. conserved neighbourhood). Another ~45% had at least one ortholog with conserved regulation and at least one other ortholog with either non-conserved regulation (making up 20 percentage points) or diverged regulation (making up the remaining 25 percentage points). The last ~10% of the genes had at least one ortholog with diverged regulation or orthologs with neither conserved nor diverged regulation. Thus ~90% of the genes with more than one predicted ortholog had conserved regulation and ~30% had diverged regulation, which in both cases are considerably higher than in the full set of genes (Figure 6B versus Figure 5A).

#### Regulation versus sequence divergence

**Figure 7:**
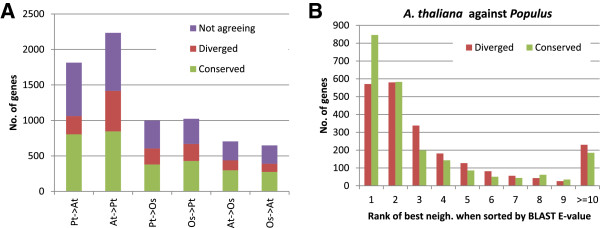
**Network neighbourhood scores versus sequence similarity. (A)** In each comparison X ─ > Y, we only consider the subsets of genes in X with at least one conserved and at least one diverged ortholog-neighbourhood in Y (blue parts of the bars in Figure 6). These genes are divided into genes where the most sequence similar ortholog also has the most conserved neighbourhood (Conserved), the most diverged neighbourhood (Diverged) or neither (Not agreeing). **(B)** A closer look at the ranks of the most significant ortholog-neighbourhood when sorted by sequence similarity.

We also compared regulation and sequence divergence for genes with more than one predicted ortholog. We found that for only ~45% of the genes that had at least one ortholog with conserved gene regulation did the ortholog with the *most* conserved regulation (i.e. highest neighbourhood overlap) also have the highest sequence similarity (i.e. BLAST bit-score). For genes that had at least one ortholog with diverged regulation, the ortholog with the *most* diverged regulation (i.e. lowest neighbourhood overlap) had the highest sequence similarity in ~25% of the cases; almost exactly what one would expect by chance given the number of predicted orthologs between these species. Since genes with conserved neighbourhoods and genes with diverged neighbourhoods are different subsets of genes, we also investigated the overlapping set of genes with both conserved and diverged orthologs (Figure 7). The same observation was made here: The most sequence similar ortholog was the ortholog with the most conserved gene regulation in ~41% of the cases and the most diverged in ~21% of the cases.

#### Reciprocal network neighbourhood comparison

**Figure 8:**
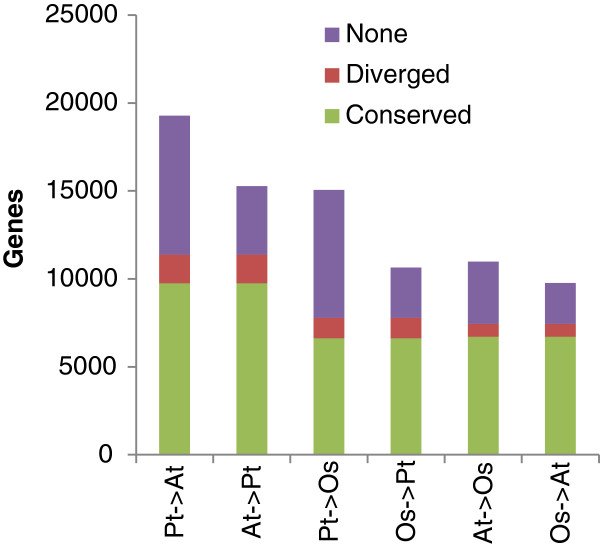
**Reciprocal network neighbourhood comparison.** Reciprocal pairwise comparisons of network neighbourhoods at a CLR threshold of two. In each comparison X ─ > Y, the genes in species X is divided into genes with a reciprocally conserved ortholog-neighbourhood in species Y (Conserved), genes with a reciprocally diverged neighbourhood in Y (Diverged) and genes with no reciprocally significant neighbourhoods in Y (None).

We also looked at network comparisons where a neighbourhood was considered conserved/diverged only if it was the most significant neighbourhood reciprocally between the gene in one species and the ortholog in the other species. Taking such an approach at a CLR threshold of two, conservation levels dropped from ~80% (Figure 5) to ~40-70% (Figure 8) depending on the species compared. Reciprocal comparisons particularly reduced neighbourhood conservation in *Populus*; *Populus* has two highly similar “versions” of many genes, and reciprocal comparisons only allow one of them to be conserved/diverged. Despite this, there were ~7,000-10,000 reciprocally conserved genes across the three species.

### ComPlEx case studies: genes with multiple ortholog candidates

**Figure 9:**
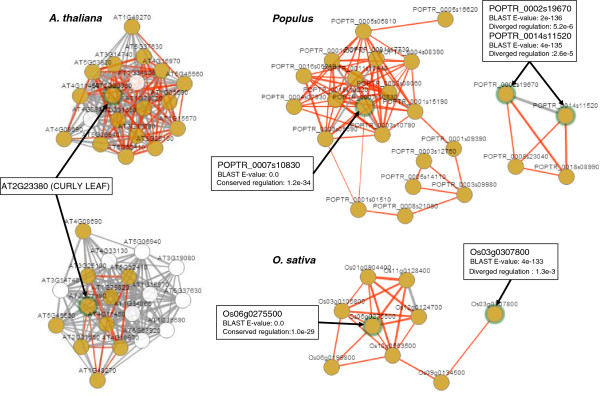
**ComPlEx case study.** The network neighbourhood of AT2G23380 (CURLY LEAF) in *A. thaliana* (CLR threshold of 3.5) and the corresponding networks of all connected orthologs in *Populus* and *O. sativa*. Red links are conserved and are drawn for a CLR threshold of two. The orthologs of AT2G23380 are marked by green auras and by arrows.

ComPlEx visualizes conserved link in co-expression networks across pairs of species. Gene lists for the comparison can be provided directly or by searching for gene IDs, GO annotations or other keywords in the database. ComPlEx allows dynamic manipulation of the networks including relocating nodes, removing nodes (for example unconnected genes) and adding co-expressed genes at any CLR threshold. As an example, we looked at the co-expression neighbourhood of the transcription factor CURLY LEAF (AT2G23380, Histone-lysine N-methyltransferase). Fittingly, the neighbourhoods were enriched for genes involved in *histone lysine methylation* (FDR corrected p-value of 1e-05 [43]). We used ComPlEx to show that CURLY LEAF has three orthologs in *Populus*, one with conserved and two with diverged regulation, and two orthologs in *O. sativa*, one with conserved and one with diverged regulation (Figure 9). Furthermore, the tool provides network statistics showing for example that the genes with the highest betweenness centrality in both the *Populus* and the *O. sativa* subnetworks (POPTR_0001s15190 and Os10g0563500, respectively) were the genes that connected the densely linked subnetwork (containing the conserved ortholog) with a more sparsely linked part of the network.

**Figure 10:**
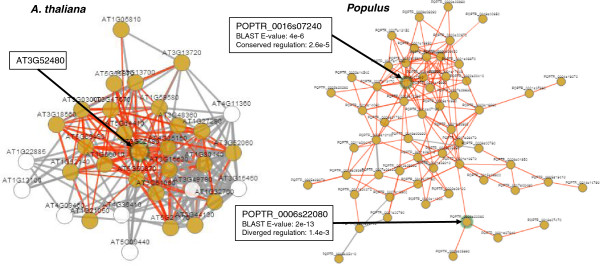
**ComPlEx case study.** The network neighbourhood of AT3G52480 in *A. thaliana* (CLR threshold of five) and the corresponding network of all connected orthologs in *Populus*. Red links are conserved and are drawn for a CLR threshold of two. The orthologs of AT3G52480 are marked by green auras and by arrows.

In the case of CURLY LEAF, the orthologs with conserved regulation were also the most sequence similar orthologs in both *Populus* and *O. sativa*. However, as we have shown in this article, this is generally not the case. We looked at AT3G52480 as an example. Interestingly, the network neighbourhood of this uncharacterized gene was enriched for genes involved in *response to fructose stimulus* (FDR corrected p-value of 5.5e-06). We used ComPlEx to show that this gene has two orthologs in *Populus*; the most sequence similar ortholog candidate had diverged regulation, while the one with lower sequence similarity had conserved regulation (Figure 10). This is an example of an *A. thaliana* gene where the ComPlEx tools could help a biologist to select the most appropriate *Populus* gene for e.g. a knock-down study. More generally, both these case studies illustrate a particularly useful application area of ComPlEx for experimental biologists; the ability to take a single gene, draw the links to co-expressed genes (i.e. the network neighborhood) and then visualize for which ortholog candidates these links are conserved in other species.

## Discussion

Biological networks have significantly different properties from random networks and studying these properties can provide insight into the basic mechanisms of biological systems [45]. We conducted a comprehensive comparison of co-expression networks in plants including the use of network properties and motifs that had previously only been studied in *A. thaliana*[36]. We also reported results for a range of co-expression thresholds (CLR thresholds) while other studies only applied a fix threshold [25,28,29]. Our observation that scale-free characteristics in all three species emerged only gradually when the CLR threshold was raised shows that the organization of co-expression networks is highly threshold dependent. Lowering the threshold eventually led to so many false positive links that the networks lost their underlying scale-free organization. On the other hand, raising the threshold led to an increasing number of false negative links; strong co-expression links were clearly more conserved across pairs of species than weak links but they were not necessarily strong in both species (Figure 4). Thus we chose to conduct in-depth analysis of network neighbourhood conservation in large networks where the statistical power is greater (CLR threshold of two), while we chose to do our analysis of network properties and motifs in the scale-free range (CLR threshold of four).

Gene networks in combination with gene centrality measures are increasingly being applied to select interesting candidate genes for further analysis (e.g. [46]). We showed that high gene centrality was significantly conserved across the three species, indicating that networks in these plants have not only retained the same organisation (i.e. scale-freeness) but that the genes upholding this organisation also are the same. Genes with conserved high local centrality in the co-expression networks were most notably enriched for photosynthesis, and never for development processes, which corresponds with the observation that the photosynthesis subnetwork was dense while many developmental subnetworks were sparsely linked. On the other hand, the genes with the highest degree in the gene regulation networks were enriched for several developmental processes. This indicates a pattern in which processes in mature tissues, such as photosynthesis, are highly co-expressed while developing tissues display less expression similarity but are tightly regulated. Such an observation instinctively makes sense, as developmental processes must inherently be buffered from extensive environmental or other modifiers to expression to ensure correct establishment of an orgasm. In contrast, once organ/tissue identity and function has been established, the ability to adapt expression to the numerous changes that can exist in the external environment (both biotic and abiotic) becomes essential to ensure survival and healthy functioning of that organ/tissue. Finally, it is intriguing that one in ten conserved hub-gene remain of completely unknown function. These presumably essential and important genes most certainly warrant further attention from molecular biologists (Additional file 2).

In additional to gene centrality, also Gene Ontology (GO) subnetworks displayed conserved properties across the three species. Dense subnetworks was particularly conserved, almost half of the top 10% most dense subnetworks were the same across the three species, and clearly play a central role in the scale-free organization of these networks by harbouring many hub genes. Chemicals in biological systems are modified by a series of chemical reactions, and a particularly strong pattern in our analysis of subnetworks was the modular (low connectivity) nature of such pathways. Finally, protein complex assembly was the most regulated subnetwork across the species (high ingoing connectivity), suggesting the importance of timely regulation for the correct assembly of protein complexes.

Assortativity measures to what extent nodes tend to mix with similar nodes in a network. It has been claimed that biological networks tend to be disassortative, e.g. high degree nodes have low degree neighbours or, equivalently, high degree nodes have low average nearest neighbour (avnn) degree [47], but different combinations of (dis) assortative biological networks with (dis) assortative hubs have also been observed (local assortativity) [48]. We observed that our co-expression networks were in general assortative (Figure 2B) and that genes with conserved high degree and avnn degree where enriched for the same biological processes. This is somewhat expected since co-expression networks are highly transitive; two co-expressed genes will often also be co-expressed with many of the same other genes. It is therefore intriguing that we also saw clear evidence of *local* disassortativity across all three species (Figure 2B) especially in the scale-free range, and that this was associated with distinct functional categories involving binding.

Cellular systems are believed to be modular where specific patterns of connected genes (motifs) are used as templates to carry out distinct functions [35]. Our analysis of network motifs seems to support this hypothesis as different motifs were often enriched in GO subnetworks of quite different biological function. We only investigated motifs of three and four genes because computation of higher order motifs becomes rapidly intractable and because smaller order motifs can be interpreted and recognized as e.g. the feed-forward motif or the bi-fan motif. Also, since we did not attempt to infer one-way directionality between TFs in our gene regulation networks (i.e. a directed link from TF A to TF B was always accompanied by a directed link from TF B to TF A), many theoretically possible motifs of three and four genes were not observed in our network (e.g. the motif in Figure 3A is not a proper feed-forward loop, FFL). This could be the reason why network motifs were clearly less conserved than gene centrality and network statistics. Although network properties and motifs have not previously been studied across plant species, Carrera *et al*. [36] thoroughly investigated such characteristics in a regulatory network of *A. thaliana.* Our results now offer the possibility to consolidate their findings across several species. For example, the FFL motif is associated with robustness to perturbations in individual links and was found to be highly enriched in stress responses by Carrera *et al*. Our analysis confirmed this finding in *A. thaliana* and, moreover, shows that this is conserved in *O. sativa* and *Populus.*

In addition to the comparisons of network properties and network motifs, we also conducted a direct comparison of gene-gene links and network neighbourhoods in the networks. Although statistically significant, only ~20-40% of the co-expression links were conserved in pairs of species when comparing equally sized networks (same CLR threshold). On the other hand, up to 80% of the strongest co-expression links (CLR threshold of six) were conserved when comparing to a network including weaker links (CLR threshold of two contained ~10% of all possible links) in the other species. This clearly demonstrates that stronger co-expression is more conserved than weaker co-expression. It has been suggested that low gene-gene link conservation in biological networks is due to a large numbers of non-essential (neutral) interactions [23]; in the same way that most fixed mutations are neutral in genomic evolution we may hypothesize that most changes to links in co-expression networks are also neutral. We thus also compared network neighbourhoods of each gene statistically, and found that a staggering ~80% of genes had a significant number of conserved co-expression partners (CLR threshold of two). Thus, even though there were relatively few conserved co-expression links in the network they were numerous enough to constitute a significant enrichment in most neighbourhoods. These findings are in agreement with those of Mohavedi *et al*. [28] where 77% of *A. thaliana* – *O. sativa* orthologs had conserved co-expression network neighbourhoods even though only a minority (~10-45%) of the genes in these neighbourhoods were orthologous. Since they studied 1:1 orthologs, the observed fraction of genes with diverged neighbourhoods were expectedly lower than what we report (8.5% versus ~20%).

A somewhat surprising trend in the network comparisons was that as the CLR threshold increased, conservation dropped (Figure 4). For link comparisons, the trend was likely caused by the networks becoming sparser and thus the *a priori* chance of a link being conserved decreased. This is supported by the observation that strong co-expression were indeed more conserved than weak co-expression when compared to a network of the same size. For network neighbourhood comparisons, the trend was even stronger, and was likely due to the increasing statistical power when comparing larger neighbourhoods. This is also why we did not see neighbourhood divergence for high thresholds; small neighbourhoods were typically not significantly non-overlapping even when they did not overlap at all. In addition to the size of the compared networks, the number of predicted orthologs also affected the level of conservation. Due to a recent genome duplication in *Populus*[49], *A. thaliana* and *O. sativa* genes have more predicted orthologs in *Populus* and hence obtained higher conservation levels when compared to *Populus* than to each other (Figure 4). Again this was likely due to the higher *a priori* chance of a link being conserved when multiple orthologs existed and indeed this trend was also observed for all species pairs when comparing exclusively genes with multiple orthologs (Figure 6). Interestingly, links in the gene regulation network were consistently less conserved than links in the co-expression network; up to ten percentage points for the *A. thaliana – O. sativa* comparison (Figure 4). Although this trend was less pronounced for conservation of gene centrality and there was no corresponding trend for network neighbourhoods (perhaps because there were enough conserved links in most neighbourhoods to make them significant anyway) this might reflect the role of regulatory networks as the driving force behind species divergence. It has previously been shown that gene regulation networks consistently rewire at higher rates than other biological networks including protein interaction networks and metabolic pathways [50].

Our analyses of network property, motif and link/neighbourhood conservation across pairs of species did not clearly reflect the phylogenetic relationship between the three plants; *A. thaliana* and *Populus* did not show notably higher conservation to each other than they did to *O. sativa* (Additional file 2, Additional file 3 and Additional file 4, Figure 4). However, the actual number of genes with conserved neighbourhoods was higher between *A. thaliana* and *Populus* (>11,000) than for *O. sativa* comparisons (<10,000, Figure 5A). This was particularly true for the reciprocal comparisons where almost 10,000 genes were conserved between *A. thaliana* and *Populus* compared to less than 7,000 genes in comparisons involving *O. sativa* (Figure 8). Taken together with the number of genes with conserved neighborhoods across all three species (~8,000, Figure 5B), this demonstrates the truly genome-wide nature of our study. For example, Ficklin *et al.* aligned only 1,173 gene loci between *Z. mays* and *O. sativa*[29] and Mohavedi *et al*. [28] compared the network neighbourhoods of 4,630 1:1 *A. thaliana – O. sativa* gene pairs.

Gene duplication followed by subfunctionalization is an important form of gene evolution [32]. Our approach allowed us to quantify, in the context of species comparison, to what extent genes have evolve through gene duplication, manifested by multiple predicted orthologs for a gene, followed by *regulatory subfunctionalization*[51], manifested by gene having at least one ortholog with conserved regulation and at least one other ortholog with a non-conserved/diverged regulation (Figure 6). We found that almost half of the genes with multiple orthologs (~45% depending on the species pair) where associated with regulatory subfunctionalization. More strictly, about one-quarter were associated with regulatory neofunctionalization, that is, one of the non-conserved orthologs had acquired completely new co-expression neighbours (i.e. had significantly diverged). Interestingly, *protein modification* was the biological process most strongly associated with regulatory subfunctionalization across all three species (Additional file 5: Figure S2), potentially indicating that such modifications play an important role in species differentiation.

Large families of closely related sequences make ortholog prediction in plants particularly challenging; predictions from OrthoMCL contained from two to four orthologs per gene on average depending on the species pair. Our results showed that, of the genes with multiple predicted orthologs, the most sequence similar ortholog was *not* the ortholog with the most conserved gene regulation (i.e. network neighbourhood) in well over half the cases (Figure 7). This demonstrates that relying on sequence similarity alone might identify an ortholog with the correct molecular function (using the GO vocabulary), but will more often than not fail to identify an ortholog that participates in the correct biological process. Thus taking into account both sequence and expression is of outmost importance when using e.g. *A. thaliana* genes to select targets in non-model organism. Patel *et al*. [11] found that the most sequence similar ortholog was *not* the expressolog (ortholog with the most correlated expression profiles across equivalent tissues) in 18-39% of the comparisons. That study thus indicate a less dramatic divergence of regulation and sequence than what we report here, however, their study was based on direct comparisons of expression changes in equivalent tissues while our analysis was based on comparisons of co-expression partners inferred across tissues *and* conditions. Hence, this might indicate that tissue specific expression of paralogs has diverged less than that of condition specific expression.

A major research challenge that has received considerable attention in recent years is the development of computational methods to reverse engineer regulatory network from gene expression data [52,53]. Due to the complexity of more advanced inference methods, genome-wide network inference is generally reduced to applying one of several measures of statistical dependency to compute pairwise correlations and to construct networks by linking genes with a correlation above a certain threshold (i.e. co-expression networks) [54]. Mutual information (MI) is a correlation measure that has gained widespread recognition due to its non-parametric nature and its robustness to outliers. The CLR algorithm is a local background correction method that has been shown to eliminate false positive correlations and indirect dependencies in co-expression networks [38]. For each pair of genes, the method computes a Z-score using a null distribution obtained from the scores between these two genes and all other genes. In our analysis, we compared networks with a Z-score threshold of two and upwards. When studying alternative correlation measures, we found that these measures disagreed primarily in the presence of outliers and that mutual information offered a robust compromise between Pearson (strongly affected by outliers) and Spearman (largely disregarding outliers). We also found that CLR highlighted the relative co-expression similarity of genes and resulted in more stable and meaningful comparisons when using the same threshold across species.

A concern when studying co-expression networks inferred across different tissues is that tissue specificity is a dominant driver of co-expression i.e. that photosynthesis genes are co-expressed simply because these genes are highly expressed in leaves and lowly expressed in other tissues. By studying tissue specificity using the tau score [28], we found that many genes that are thought of as tissue specific, including the photosynthesis genes, are in fact co-expressed within a number of tissues and that expression similarity in our networks is mainly driven by co-expression within tissues and not differential expression between tissues (see Additional file 6: Figure S3A). One might speculate that this tendency of universal co-expression is the reason why we observed such high levels of network neighbourhood conservation across species even though we compared expression data from a heterogeneous set of experiments. It is rather striking that despite the fact that we used all available experiments without filtering for congruence, we observed significant gene regulation conservation for around 80% of the genes. Nonetheless, more homogenous datasets would almost certainly have resulted in higher conservation, at least higher link conservation. Relatedly, some of the differences between co-expression networks may stem from noise in microarray data related to cross hybridization and the fact that the *Populus* data come from multiple species within the genus. Sequence-similar paralogs are known to be associated with particularly unreliable microarray-based expression profiles due to cross-hybridization [55]. This could in particular have affected our comparison of paralogous network neighbourhoods. Although we did not observed noticeable artefacts when comparing the expression of paralogs with conserved and diverged network neighbourhoods (Additional file 6: Figure S3B), we cannot rule out false negative network neighbourhood divergences that were lost due to averaging of expression across paralogs. Hence we might have underestimated the divergence of paralog regulation.

## Conclusions

Both the data resources and computational methods are now available to take the step from sequence-based comparative genomics to transcriptomics-based comparative regulomics. We conducted the first comprehensive comparison of the co-expression networks of *A. thaliana*, *Populus* and *O. sativa* that included network properties and motifs as well as gene-gene links and network neighbourhoods; at different co-expression thresholds.

The organization of the networks changed from near random networks at low CLR thresholds to scale-free networks at higher threshold. The most central genes in this organization were conserved. Many interesting conserved properties could be observed in Gene Ontology (GO) subnetworks such as, for example, that metabolic subnetworks tended to be modular and sparsely connected to the rest of the network and that network motifs seemed to act as templates for realizing specific functions. We identified two particularly interesting motifs that were enriched in GO subnetworks associated with response to stimuli and flower development, respectively, in all three species.

At the level of individual gene-gene links, the networks were highly diverged (only 30-40% similar), while at the level of network neighbourhoods they were largely conserved (~80% similar). This could be because most individual links are non-essential, and that the underlying conservation only emerges as statistically significant at the neighbourhood level. We found that about half of the genes with more than one predicted ortholog had at least one ortholog with conserved regulation and at least one ortholog where the regulation was either diverged or at least not conserved. These findings go some way to quantify the level of regulatory subfunctionalization that occurred in these species. Interestingly, the most sequence similar predicted ortholog was *not* the ortholog with the most conserved regulation in over half of the cases (55-60%). We have shown how one can use sequence to predict candidate orthologs, and then use network neighbourhood conservation to select the most appropriate ortholog. And we have provided a web portal, ComPlEx, making this type of analysis readily accessible to molecular biologists.

## Methods

### Data

We downloaded all Affymetrix experiments for *A. thaliana* (6665 experiments), *Populus* (462 experiments from multiple Populus species) and *O. sativa* (711 experiments) from the Gene Expression Omnibus (GEO) (data downloaded March 2011). Raw data were normalized using the RMA normalisation method as implemented in the Bioconductor [56] package ‘affy’ [57] for the R statistical language using default settings. Expression data from the different expression datasets for each species were merged using the MergeMaid (http://astor.som.jhmi.edu/MergeMaid) package.

Orthologs were predicted using OrthoMCL with default parameters [41]. BLAST Bit-scores were used to rank paralogs in the same OrthoMCL group (to resolve ties when E-values were zero). We used the annotated transcription factors available from PlantTFDB [58]. We downloaded the latest TAIR [59] Gene Ontology annotation file and transferred annotation to *Populus* and *O. sativa* trough the orthologs.

### Network inference

We computed mutual information for all gene pairs using a B-spline estimator [60] with a C-program that was adapted from Carrera *et al.*[36]. The number of bins used to compute the probabilistic profiles for the expression of each gene was chosen in the range of the square of the number of samples (as recommended by the authors). We then use the CLR method to background-correct the MI values and obtain edge-vicinity Z-scores (called CLR scores) [38,39]. While the MI score are in the [0, 1] interval, the CLR scores are fat-tail distributed in the [0, 30] interval. We applied CLR thresholds between 1 and 10 and performed our analysis on the resulting networks. We looked at two distinct types of networks: gene-gene co-expression networks that were the direct result of applying a threshold on all the CLR scores and transcription factor – gene (TF-gene) regulation networks that were filtered using the set of annotated TFs. Thresholds lower than three were often impractical for a number of network analyses that require intense computation, and were therefore avoided. The computations were done on several resources including personal computers, academic clusters and commercial clouds.

### Network properties

Both network statistics and motif analysis were performed with a combination of Python and R scripts using the igraph library for complex network research [61] and the scipy python library for scientific computing [62]. For the gene centrality analysis, we selected the top 10% most/least central genes in each species and counted the number of genes with predicted orthologs in the top 10% in the other species. To indicate significance, we computed p-values by counting the number of conserved genes in randomized gene lists. A corresponding approach was used to investigate conservation of network statistics and network motifs in GO subnetworks; for each statistics/motif we compared the number of subnetworks that ranked in the top 10% in all three species to the corresponding number in randomized lists. We only considered GO subnetwork with between 10 and 1000 connected genes.

Motifs of size three and four were computed for the gene regulation networks, and then recomputed for 100 randomized networks with the same degree distribution [63]. We defined significant network motifs as motifs that occurred significantly more often in the inferred networks than in the randomized networks, and ranked these motifs by their Z-score distance to the randomized tests.

### ComPlEx website

We did a lot of the analysis through our website (http://complex.plantgenie.org/). Most of the network statistics and motif scripts are active on the ComPlEx website and can be used to compute many different statistics for custom selections of genes and on a wide range of co-expression thresholds. ComPlEx has functionality that extends beyond the scope of this text and was created as a tool for comparative analysis and exploration of plant co-expression. It is built with modern html5 technology while the server runs a combination of Python and PHP.

### Network comparison

When comparing species S1 to species S2, conservation statistics was reported relative to the genes that were connected (i.e. had at least one neighbour) at a particular CLR threshold in species S1 *and* that had at least one predicted ortholog that were connected in species S2. A link between genes *A* and *B* in S1 was considered conserved if there existed a link in S2 between one of the orthologs of *A* and one of the orthologs of *B*. The fractions of conserved links were compared to fractions obtained from comparing randomized networks; 100 randomized networks were generated for each species by shuffling the gene names in the original co-expression networks. Randomized networks never obtain conservation fractions as high as the fractions obtained from the original co-expression network for any CLR threshold.

A gene *A* in S1 was considered to have a conserved neighbourhood if there existed an ortholog *A’* in S2 with a statistically significantly overlapping neighbourhood. The neighbours of *A’* in S2 were mapped back to S1 through orthologs, and a p-value was computed in S1 using the hypergeometric distribution; *N* = the number of genes in S1, *n* = the number of genes in the neighbourhood of *A*, *k* = the number of orthologs of the neighbours of *A’* and *x* = the number of genes in both the neighbourhood of *A* and the mapped neighbourhood of *A’*. A p-value was computed for each ortholog pair and statistical significance was determined using the FDR multiple hypothesis correction controlled at 0.05. We reported the fraction of genes in S1 with a statistically significant neighbourhood overlap to at least one ortholog in S2 using the FDR threshold. Significant neighbourhood divergence was computed correspondingly. We also used randomized networks to compute alternative significance thresholds, and this approach resulted in thresholds very close to the FDR thresholds but at a much higher computational cost.

## Competing interests

The authors declare that they have no competing interests.

## Authors’ contributions

SN carried out the network inference and analysis, designed and implemented the web tool and helped to draft the manuscript. DS helped implementing the web tool. NRS collected and normalized the microarray data and helped with the design and interpretation. TRH conceived, designed and coordinated the study, interpreted results and drafted the manuscript. All authors read and approved the final manuscript.

## Supplementary Material

Additional file 1: Figure S1 Ortholog predictions. The average number of predicted orthologs when comparing one species X to another species Y (X ─ > Y), where At is *A. thaliana*, Pt is *Populus* and Os is *O. sativa*. Click here for file

Additional file 2 Gene centrality conservation. Click here for file

Additional file 3 Conservation of network statistics in Gene Ontology subnetworks. Click here for file

Additional file 4 Conservation of network motifs in Gene Ontology subnetworks. Click here for file

Additional file 5: Figure S2 Fraction of conserved, diverged and conserved-and-diverged genes distributed across selected GO terms. The “all” bar correspond to the At ─ > Pt, Os bar in Figure 5B, while the other bars are the “all” bar distributed across GO biological processes from plant slim. P-values indicating enrichment of conserved, diverged, and conserved-and-diverged genes in the different GO categories are given in three columns to the right (Hypergeometric distribution and Bonferroni correction, the background is the 10 692 connected *A. thaliana* genes with connected orthologs in both the two other species). Click here for file

Additional file 6: Figure S3 Co-expression and paralogs. (A) The expression of two transcription factors associated with leaf length in some selected tissues. YAB1 (AT2G45190) is involved in abaxial cell type specification in leaves and fruits and HB22 (AT4G24660) is involved in embryo development. Although having tissue specific functional roles, the two genes were highly co-expressed not only in a number of relevant tissues but also (albeit at somewhat lower expression levels) in roots. (B) The average expression of the *Populus* ortholog with the most diverged network neighbourhoods against the average expression of the *Populus* ortholog with the most conserved network neighbourhoods for 2234 *A. thaliana* genes that are both conserved and diverged when compared to *Populus* (“Conserved and Diverged”-part of the At ─ > Pt bar in Figure 6B). We observe no noticeable artifacts such as genes with diverged neighbourhoods being lowly expressed. Also, the correlations between the most conserved and the most diverged orthologs showed reasonably dissimilar expression profiles; the correlations were reasonably normally distributed with a mean correlation of only 0.15 and 74% of the data within one standard deviation of 0.25 (i.e. within the correlation interval [−0.1, 0.4]). Click here for file
