# Supplementary figures and images for: ComPlEx: conservation and divergence of co-expression networks in A. thaliana, Populus and O. sativa
Source: BMC Genomics. 2014 Feb 6;15:106. doi: 10.1186/1471-2164-15-106 (PMC3925997; doi:10.1186/1471-2164-15-106)

## Slide 1
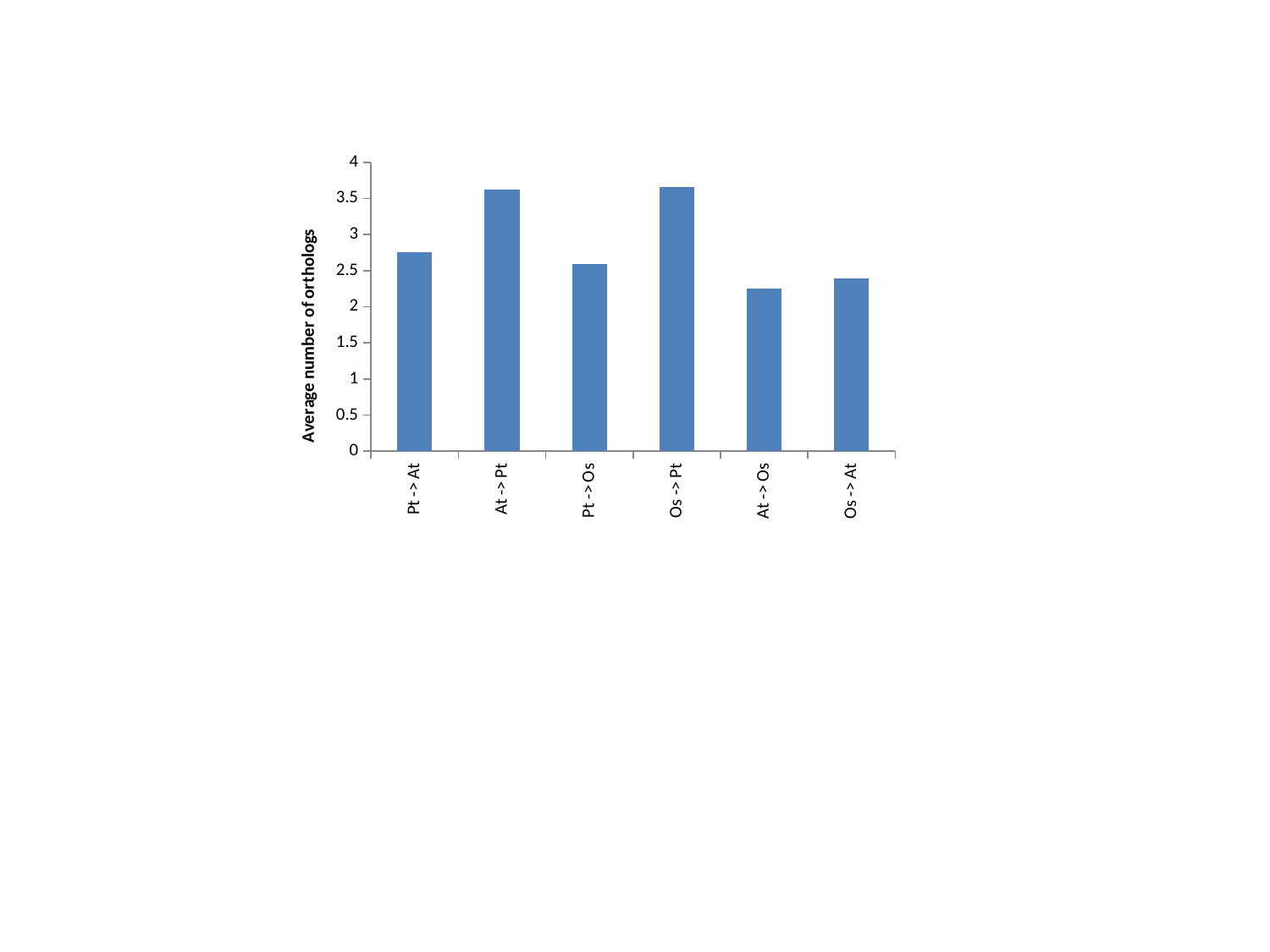

### Chart
| Category | |
|---|---|
| Pt -> At | 2.7609042107029 |
| At -> Pt | 3.62525469464178 |
| Pt -> Os | 2.59275818021028 |
| Os -> Pt | 3.65844020797227 |
| At -> Os | 2.24691274257023 |
| Os -> At | 2.39460553908453 |

Supplement: Additional file 1: Figure S1 — Ortholog predictions. The average number of predicted orthologs when comparing one species X to another species Y (X ─ > Y), where At is A. thaliana, Pt is Populus and Os is O. sativa. [file 1471-2164-15-106-S1.pptx]

## Slide 1
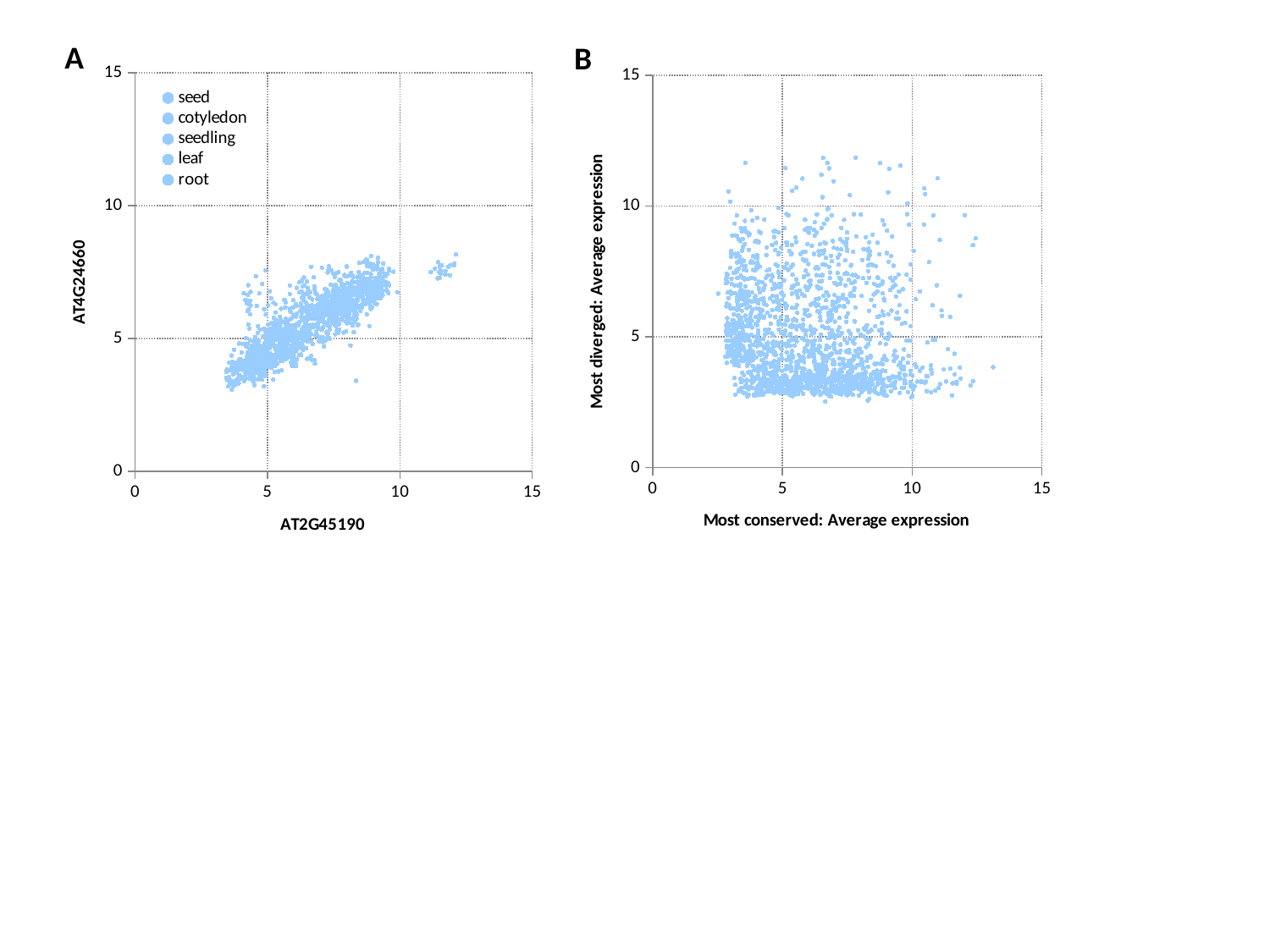

A
B
### Chart
| Category | seed | cotyledon | seedling | leaf | root |
|---|---|---|---|---|---|
### Chart
| Category | Diverged |
|---|---|

Supplement: Additional file 6: Figure S3 — Co-expression and paralogs. (A) The expression of two transcription factors associated with leaf length in some selected tissues. YAB1 (AT2G45190) is involved in abaxial cell type specification in leaves and fruits and HB22 (AT4G24660) is involved in embryo development. Although having tissue specific functional roles, the two genes were highly co-expressed not only in a number of relevant tissues but also (albeit at somewhat lower expression levels) in roots. (B) The average expression of the Populus ortholog with the most diverged network neighbourhoods against the average expression of the Populus ortholog with the most conserved network neighbourhoods for 2234 A. thaliana genes that are both conserved and diverged when compared to Populus (“Conserved and Diverged”-part of the At ─ > Pt bar in Figure 6B). We observe no noticeable artifacts such as genes with diverged neighbourhoods being lowly expressed. Also, the correlations between the most conserved and the most diverged orthologs showed reasonably dissimilar expression profiles; the correlations were reasonably normally distributed with a mean correlation of only 0.15 and 74% of the data within one standard deviation of 0.25 (i.e. within the correlation interval [−0.1, 0.4]). [file 1471-2164-15-106-S6.pptx]
