# Supplementary material for: ComPlEx: conservation and divergence of co-expression networks in A. thaliana, Populus and O. sativa
Source: BMC Genomics. 2014 Feb 6;15:106. doi: 10.1186/1471-2164-15-106 (PMC3925997; doi:10.1186/1471-2164-15-106)
Supplement: Additional file 5: Figure S2 — Fraction of conserved, diverged and conserved-and-diverged genes distributed across selected GO terms. The “all” bar correspond to the At ─ > Pt, Os bar in Figure 5B, while the other bars are the “all” bar distributed across GO biological processes from plant slim. P-values indicating enrichment of conserved, diverged, and conserved-and-diverged genes in the different GO categories are given in three columns to the right (Hypergeometric distribution and Bonferroni correction, the background is the 10 692 connected A. thaliana genes with connected orthologs in both the two other species). [file 1471-2164-15-106-S5.pptx]

## Slide 1
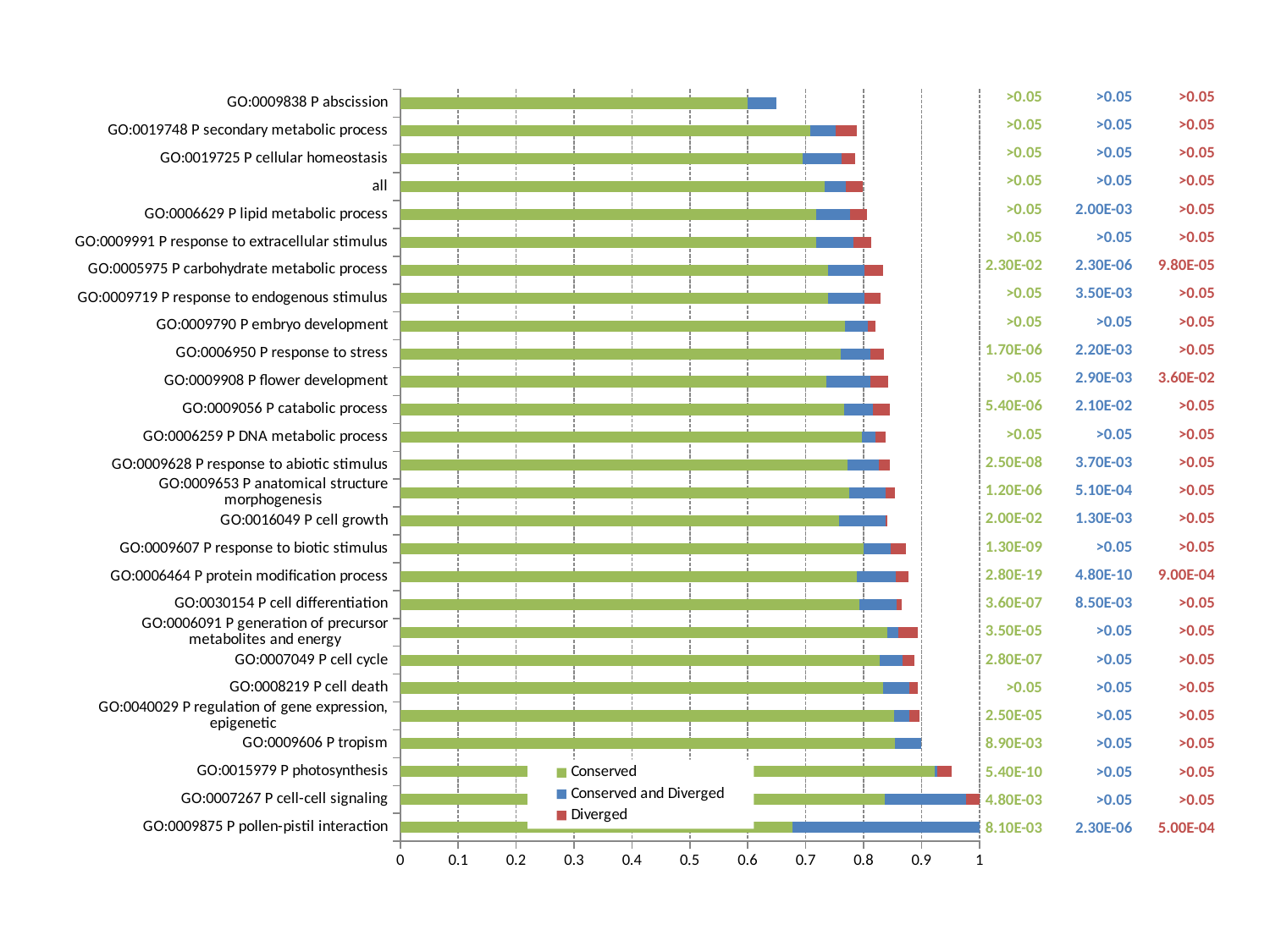

>0.05
>0.05
>0.05
>0.05
>0.05
>0.05
2.30E-02
>0.05
>0.05
1.70E-06
>0.05
5.40E-06
>0.05
2.50E-08
1.20E-06
2.00E-02
1.30E-09
2.80E-19
3.60E-07
3.50E-05
2.80E-07
>0.05
2.50E-05
8.90E-03
5.40E-10
4.80E-03
8.10E-03
>0.05
>0.05
>0.05
>0.05
2.00E-03
>0.05
2.30E-06
3.50E-03
>0.05
2.20E-03
2.90E-03
2.10E-02
>0.05
3.70E-03
5.10E-04
1.30E-03
>0.05
4.80E-10
8.50E-03
>0.05
>0.05
>0.05
>0.05
>0.05
>0.05
>0.05
2.30E-06
>0.05
>0.05
>0.05
>0.05
>0.05
>0.05
9.80E-05
>0.05
>0.05
>0.05
3.60E-02
>0.05
>0.05
>0.05
>0.05
>0.05
>0.05
9.00E-04
>0.05
>0.05
>0.05
>0.05
>0.05
>0.05
>0.05
>0.05
5.00E-04
### Chart
| Category | | | |
|---|---|---|---|
| GO:0009875 P pollen-pistil interaction | 0.67741935483871 | 0.32258064516129 | 0.0 |
| GO:0007267 P cell-cell signaling | 0.837209302325581 | 0.13953488372093 | 0.0232558139534884 |
| GO:0015979 P photosynthesis | 0.923387096774194 | 0.00403225806451613 | 0.0241935483870968 |
| GO:0009606 P tropism | 0.854545454545454 | 0.0454545454545455 | 0.0 |
| GO:0040029 P regulation of gene expression, epigenetic | 0.852233676975945 | 0.0274914089347079 | 0.0171821305841924 |
| GO:0008219 P cell death | 0.833333333333333 | 0.0454545454545455 | 0.0151515151515152 |
| GO:0007049 P cell cycle | 0.827655310621243 | 0.0400801603206413 | 0.0200400801603206 |
| GO:0006091 P generation of precursor metabolites and energy | 0.841232227488152 | 0.018957345971564 | 0.033175355450237 |
| GO:0030154 P cell differentiation | 0.792604501607717 | 0.0643086816720257 | 0.00964630225080386 |
| GO:0006464 P protein modification process | 0.787916928886092 | 0.0679672750157332 | 0.0220264317180617 |
| GO:0009607 P response to biotic stimulus | 0.79960899315738 | 0.0478983382209189 | 0.0254154447702835 |
| GO:0016049 P cell growth | 0.756983240223464 | 0.0810055865921788 | 0.00279329608938547 |
| GO:0009653 P anatomical structure morphogenesis | 0.77457264957265 | 0.063034188034188 | 0.0170940170940171 |
| GO:0009628 P response to abiotic stimulus | 0.772839506172839 | 0.0530864197530864 | 0.0191358024691358 |
| GO:0006259 P DNA metabolic process | 0.797777777777778 | 0.0222222222222222 | 0.0177777777777778 |
| GO:0009056 P catabolic process | 0.765755053507729 | 0.0505350772889417 | 0.0291319857312723 |
| GO:0009908 P flower development | 0.736040609137056 | 0.0761421319796954 | 0.0304568527918782 |
| GO:0006950 P response to stress | 0.760839160839161 | 0.0508158508158508 | 0.0233100233100233 |
| GO:0009790 P embryo development | 0.767810026385224 | 0.0395778364116095 | 0.0131926121372032 |
| GO:0009719 P response to endogenous stimulus | 0.739187418086501 | 0.0629095674967235 | 0.0275229357798165 |
| GO:0005975 P carbohydrate metabolic process | 0.738862234407128 | 0.0623714873200822 | 0.0322138450993831 |
| GO:0009991 P response to extracellular stimulus | 0.718085106382979 | 0.0638297872340425 | 0.0319148936170213 |
| GO:0006629 P lipid metabolic process | 0.718428437792329 | 0.058933582787652 | 0.028999064546305 |
| all | 0.732697343808455 | 0.0363823419378975 | 0.0294612794612795 |
| GO:0019725 P cellular homeostasis | 0.695238095238095 | 0.0666666666666667 | 0.0238095238095238 |
| GO:0019748 P secondary metabolic process | 0.708126036484245 | 0.0431177446102819 | 0.0364842454394693 |
| GO:0009838 P abscission | 0.6 | 0.05 | 0.0 |
